# Scientometrics on Public Health Research in Iran: Increase of Area Studies despite Embargoes? A Review Article

**Published:** 2017-03

**Authors:** Brice POREAU

**Affiliations:** Children Hospital, CHU Grenoble, Grenoble, France Public Health – International Studies Institute, Lyon, France

**Keywords:** Iran, Public health, Network analysis, Bibliometrics

## Abstract

**Background:**

Due to embargoes and sanctions from 1979 until 2015, impact on scientific research in Iran may be critical. Public health is the main example of this burning point. In this paper, the aim was to map the scientific research in public health in Iran until 2014 with area studies as well as networks of countries involved.

**Methods:**

We used bibliographic analyses using VOS viewer software for network analysis during the period 1975–2014. Two databases were used: Web of Science and PubMed. We performed analyses of journals, authors, publication years, organizations, funding companies, countries, keywords and Web of sciences Categories.

**Results:**

We accessed 862 articles published between 1991 and 2014, the majority of published after 2008. The main countries of research were Iran, the United States of America, England, and Sweden and represented the main network collaboration. The main Web of Sciences categories was public, occupational and environmental health, medicine general internal and parasitology. We accessed 25462 publications on PubMed database from 1950 to 2014. The majority of published after 2004. The main area studies were prognosis, wounds and injuries, soil solutions and biological markers.

**Conclusion:**

Public health research in Iran has been developed since 2004. The chief field was emerging cardiovascular diseases and communicable diseases. Other biotechnological fields were emerging such as biological markers research. Iran provides structures to face up with its new challenges using networks of countries such as the USA, England, and Sweden. End of embargoes could provide new perspectives for public health research and more largely scientific research in Iran.

## Introduction

In recent years, despite embargoes from 1979 until 2015, Iran has made significant efforts at facing up a number of health development challenges. Modernization of health system has been a major goal (1) and progress toward improving health equity has been significant (1). Cooperation between the Ministry of Health and Medical Education (MHME) and the World Health Organization (WHO) Country Office for Iran allowed main strategies for this progress in public health^1^. Public health covered the fields such of cardiovascular diseases, infectious disease, mental health, organization of the health facilities and their financing.

In 2010, WHO presented several programs on public health in Iran (1). The 2010–2014 cooperation strategy’s main objectives were improving health equity and social determinants of health, strengthening primary health care, achieving universal coverage, improving equity in health care financing, improving leadership and governance, strengthening health security, managing the demographic and epidemiological transition and strengthening partnership for development (1).

The aim of this article was to analyze scientific publications on public health in Iran during the period 1950–2014 and to expose networks involved and the emerging area studies. The method research is bibliometric using VOSviewer software for network analysis.

## Methods

We used two databases: the Science Citation Index-Expanded (SCIE) database core collection, accessed through the Web of Science (WOS) platform from Thomson Reuters and PubMed database.

In the advanced search from WOS, we obtained the articles using this formula: TS= (Iran AND public health) for the period 1975–2014. We verified each record to ensure its relevance. There were no restrictions regarding the document types.

Then, we performed the “analysis results” function of WOS. We extracted journals, most cited articles, authors, countries, funding agencies, organizations, publication years and Web of Science categories.

In order to evaluate the research networks between countries, after the analysis by country, we viewed the records of each country and then performed the analysis a second time in order to understand the links between the chosen country and the other countries. We then established the mapping diagram.

To analyze the Web of Science categories, we exported the date into a file “analyze.txt”. This file can be read by the program wc10.exe. It generated map files for VOSviewer (2–4).

These analyses were to compare with former bibliometric studies in other fields (5–6).

We used another database PubMed, to compare the first data set. We obtained publications using this formula: (Iran OR Iranian AND public health) AND (“1900”[Date - Publication]: “2014/12/31”[Date-Publication]). We extracted publication years. We extracted data with MEDLINE file. We then analyzed Medical Subject Headings (MeSH) with previously method described. We generated VOSviewer picture as described.

## Results

From WOS database, we obtained 862 records. The first publication was in 1991. There were 739 publications (85.73%) after 2008.

The three authors with most records were attached to Iranian academic institutions. The main journals were *Iranian Journal of Public Health* (6.7%), *Public Health* (3.48%), *Archives of Iranian Medicine* (3.48%), *Iranian Journal of Parasitology* (3.36%).

The main research-funding agency was the Ministry of Health and Medical Education.

The most represented institutions were the University of Tehran, the Islamic Azad University, Isfahan University, Shiraz University and Shahid Beheshti University. All these institutions are from Iran.

The ten main involved countries were Iran (86.3%), the USA (7.3%), England (3.7%), Sweden (3.2%), Canada (2.3%), Australia (2.2%), Turkey (1.6%), Finland (1.5%), Germany (1.5%) and Saudi Arabia (1.2%).

**Fig. 1:**
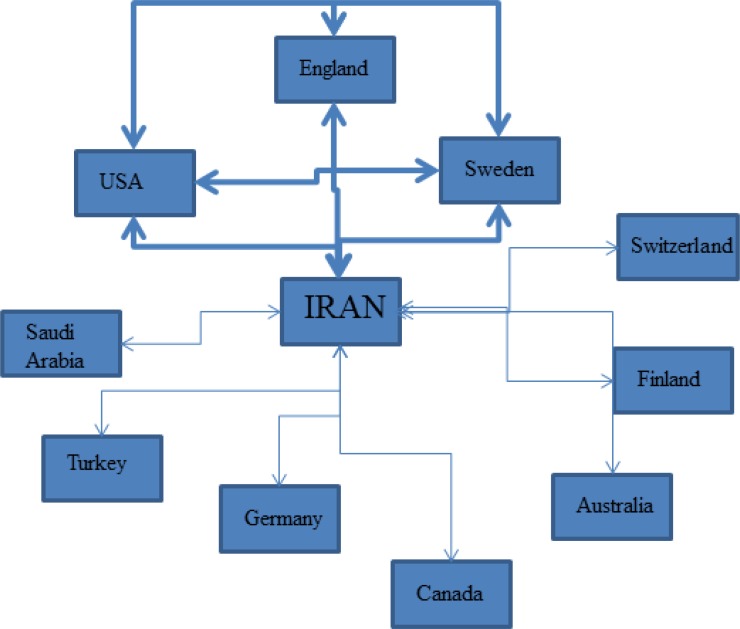
The scientific network concerning public health research of Iran with the main countries involved is described. Iran alone is the most important country. However, we revealed a second main network between Iran, the USA, England and Sweden. Finally, less important networks are represented between Iran and countries from Europe, Arabian Peninsula and Australia

We mapped the networks between the main countries (Fig. 1).

The scientific network concerning public health research of Iran with the main countries involved is described. Iran alone is the most important country. However, we revealed a second main network between Iran, the USA, England, and Sweden. Finally, less important networks are represented between Iran and countries from Europe, Asia, and Australia.

**Fig. 2:**
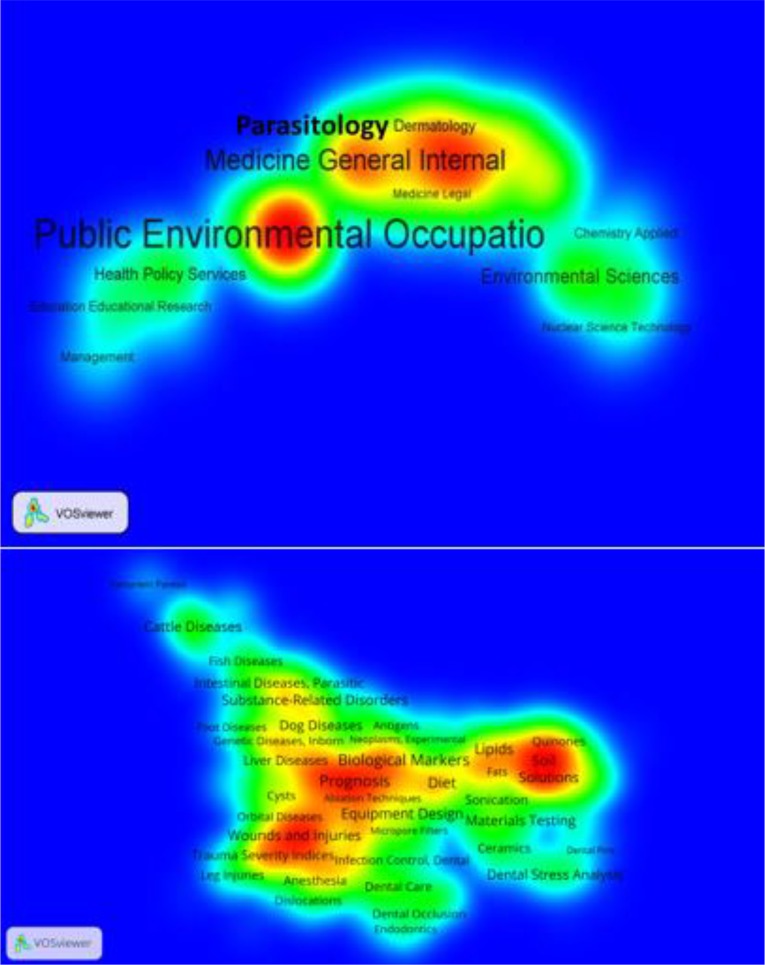
a) Web of Sciences Categories mapped with VOSviewer. The most important categories are public environmental occupation, medicine general internal and parasitology b) Pubmed area studies (Medical subject headings) mapped with VOS viewer. The most important area studies are prognosis, wounds and injuries, biological markers, soil solutions

Finally, we used VOSviewer in order to map the Web of Sciences categories (Fig. 2). The most important categories were public, environmental and occupational public health, medicine general internal and parasitology.

From PubMed database, we obtained 25462 records from 1950 to 2014. 93.74% of records were after 2004.

Using VOSviewer to map the medical subject headings, the most important area studies are prognosis, wounds, and injuries, biological markers, soil solutions. Furthermore, we noticed also other area studies such as dental care and intestinal diseases, parasitic.

## Discussion

According to our analysis on both databases (PubMed and WOS), most studies were published after 2004. The progress of publications was concordant with others studies (5–6). The increase of publications after 2004 on public health research may correspond to previous WHO medium-term strategic plan (1).

According to our study on one database (WOS core collection), public health research in Iran focuses on three main topics: communicable diseases, cardiovascular diseases and epidemiology in obesity and overweight (Fig. 2a).

Research on communicable diseases is concordant with MDG (1). Moreover, research focuses on parasitology and concerns all parts of Iran (7–9). Parasitology is a main field of research in Iran (Fig. 2a). Not only epidemiologic studies are performed, also, fundamental research (7–9).

Cardiovascular diseases are the most common etiology of mortality in Iran (10). Public health research on cardiovascular diseases is, therefore, the main issue (10–11).

The lack of physical activity is the main risk factor for cardiovascular diseases (1). That is why research on obesity and overweight is also a main topic in public health (11). Specific epidemiologic studies are performed to understand and to fight against the increasing prevalence of obesity (12–13).

Nevertheless, other topics are also relevant in the public health research in Iran, such as breast cancers for example (14). The study of the second database PubMed allows us to describe other area studies such as biological markers and prognosis, related to cancer (Fig. 2b). Furthermore, this second database analysis reveals two other different fields: environment research (soil) as well as dental care (Fig. 2b).

The analysis of only one database (WOS collection core) reduces the bibliometric study, however, the correlation with the second one (PubMed) confirms the main topics and enhances the bibliometric study.

These different topics reveal the challenges of the third populous Eastern Mediterranean country. Seen as a lower-middle income country (1), Iran has to face up with Southern country public health problems, such as parasitology, and Western countries public health problems, such as cardiovascular diseases, cancers as well as biotechnological topics with prognosis and biological markers or dental care (Fig. 2b).

To achieve the so different challenges, structures and programs are in place (15–16). Public research in Iran is mainly financed by Iran itself (Fig. 1). In fact, even if secondary networks with the USA, England, and Sweden is noticed (Fig. 1), Iran alone represents more than 85% of the records. It is confirmed by the main funding agency: the Iranian Ministry of Health and Medical Education. Only the database WOS core collection provides us with the tools to perform such analysis. More bibliometric studies could confirm this network analysis and financing system. In fact, a previous study in 2011 presented an increasing trend of publications but with research budget much more less than that of the developed countries (17). This study confirmed to potential of Iran in the field of public health research and confirms the need for a higher budget.

Finally, Iran provides structures to face up with its new so different challenges from parasitology to cardiovascular diseases, cancers, dental care or environment. Iran is related not only to the three countries involved in the main networks: England, the USA, and Sweden but in networks of all around the world: Europe and Australia (Fig. 1).

## Conclusion

Iran provides structures to face up with its new challenges using networks of countries such as the USA, England, and Sweden. The end of embargoes could provide Iran with new and enhanced networks to confirm the increase in scientific and public health research.

## Ethical considerations

Ethical issues (Including plagiarism, informed consent, misconduct, data fabrication and/or falsification, double publication and/or submission, redundancy, etc.) have been completely observed by the authors.
